# Germanium Complexes with *ONO* Tridentate Ligands: O-H Bond Activation Control According to DFT Calculations

**DOI:** 10.3390/ijms241210218

**Published:** 2023-06-16

**Authors:** Kirill V. Zaitsev, Andrey D. Trubachev, Oleg Kh. Poleshchuk

**Affiliations:** 1 Chemistry Department, M.V. Lomonosov Moscow State University, Leninskye Gory 1, 3, 119991 Moscow, Russia; zaitsev@org.chem.msu.ru (K.V.Z.); andrei.trubachev@chemistry.msu.ru (A.D.T.); 2 Faculty of Chemistry, National Research Tomsk State University, Lenin Av., 36, 634050 Tomsk, Russia; 3 Department of Chemistry, Tomsk State Pedagogical University, Kievskaya Str., 60, 634061 Tomsk, Russia

**Keywords:** Group 14 elements, hypercoordinated compounds, ligand design, *ONO* ligands, tetrylenes, tridentate ligands, DFT calculations

## Abstract

Polydentate ligands are used for thermodynamic stabilization of tetrylenes—low-valent derivatives of Group 14 elements (E = Si, Ge, Sn, Pb). This work shows by DFT calculations how the structure (the presence or absence of substituents) and type (alcoholic, Alk, or phenolic, Ar) of tridentate ligands 2,6-pyridinobis(1,2-ethanols) [^Alk^*ONO*^R^]H_2_ and 2,6-pyridinobis(1,2-phenols) [^Ar^*ONO*^R^]H_2_ (R = H, Me) may affect the reactivity or stabilization of tetrylene, indicating the unprecedented behavior of Main Group elements. This enables the unique control of the type of the occurring reaction. We found that unhindered [*ONO*^H^]H_2_ ligands predominantly led to hypercoordinated *bis*-liganded {[*ONO*^H^]}_2_Ge complexes, where an E(+2) intermediate was inserted into the ArO-H bond with subsequent H_2_ evolution. In contrast, substituted [*ONO*^Me^]H_2_ ligands gave [*ONO*^Me^]Ge: germylenes, which may be regarded as kinetic stabilized products; their transformation into E(+4) species is also thermodynamically favorable. The latter reaction is more probable for phenolic [^Ar^*ONO*]H_2_ ligands than for alcoholic [^Alk^*ONO*]H_2_. The thermodynamics and possible intermediates of the reactions were also investigated.

## 1. Introduction

In modern chemistry, selective synthesis of organometallic compounds in a determined oxidation state is an important problem. For transition metal complexes, this problem has been solved and is controlled by the oxidation state of the initial metal reagent [1,2]; for Main Group elements, the influence of organic ligand has a special role.

Tetrylenes [3,4,5,6,7]—heavier carbene analogs—have recently attracted significant attention [8,9,10,11,12,13,14,15,16,17,18,19,20]), primarily due to their possible application as analogs of transition metals in catalysis [21,22,23], in activation of small molecules [24,25,26,27,28,29,30], as ligands [31,32], in possible synthesis of derivatives with E-E bonds [33,34,35], reagents for synthesis [36,37,38,39] or precursors [40,41,42] in material chemistry. These species are reactive and unstable; to stabilize them, the introduction of bulky groups (kinetic stabilization) or polydentate ligands (thermodynamic stabilization) is used.

**Scheme 1:**
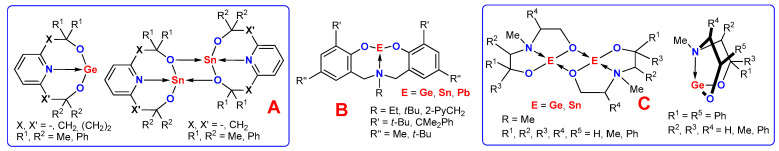
Structures of tetrylenes based on various aminoalcohols: tetrylenes based on 2,6-bis(hydroxyalkyl)pyridines (**A**); tetrylenes based on aminobis(methylenophenols) (**B**); tetrylenes based on dialkanolamines (**C**).

Different types of alkanolamines are used as such ligands. Interestingly, germylenes based on 2,6-bis(hydroxyalkyl)pyridines are monomeric, whereas stannylenes are dimeric [43] (Scheme 1; type **A**). The aggregation degree (monomer or dimer) for the related tetrylenes based on dialkanolamines is determined by the type of substituent at C and N atoms. In this case (Scheme 1; type **C**), monomeric germylenes for the voluminous ligands containing substituents at both C atoms in an α-position to O are formed; otherwise, the species formed are dimeric [44]. Stannylenes are dimeric despite the presence of substituents. Tetrylenes based on the related aminobis(methylenophenols) (type **B**) are monomeric [45]. All these data indicate that a special modification of organic structure ligand (variation of substituents at C atom, change of the type of O atom) changes tetrylenes’ aggregation degree.

**Scheme 2:**
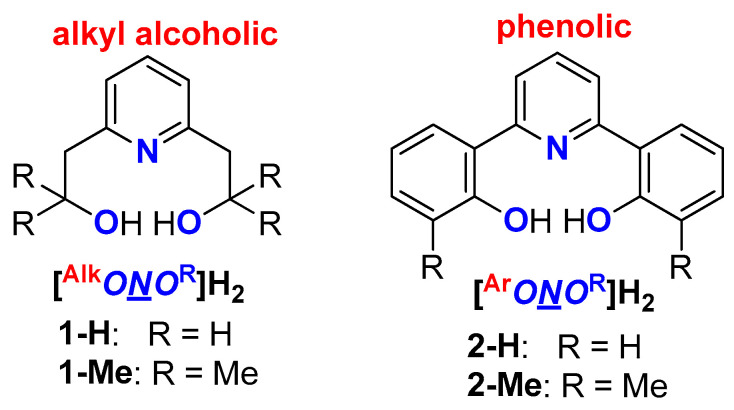
Structures of tridentate *ONO* ligands under investigation.

2,6-Pyridinobis(phenols) as ligands (here and below *ONO* ligands, where *O* atoms are connected to the metal atom by covalent bonding, and the *N* atom is coordinated by donor bonding) have been previously used to synthesize complexes of various d- and f-transition metals (Y, Al; Ti, Zr, Hf; V etc.) [46,47,48,49,50,51,52,53,54], but data on their application to Group 14 elements is scarce in the literature (see below). These phenolic ligands **2-H**, **2-Me** are analogs of alcoholic 2,6-bis(hydroxyalkyl)pyridines **1-H**, **1-Me** (Scheme 2); however, a significant modification of the donor O type is observed. Phenolic protons are more acidic, and phenolic oxygens are weaker Lewis donors.

**Scheme 3:**
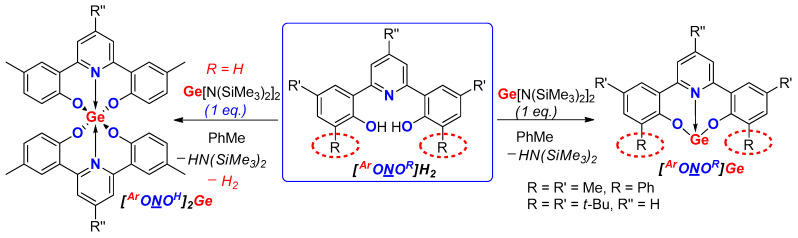
Interaction of *ONO* ligands (2,6-pyridinobis(phenols)) with Lappert’s germylene.

Interaction of tetrylenes, R_2_E: (E = Si [55,56,57], Ge [58,59], Sn [60,61]; R = Alk, Ar, OR′ etc.), with alcohols, phenols or related compounds, R’OH, has been widely used previously to trap unstable species or investigate their reactivity, resulting in the usual R_2_E(H)OR′ insertion products; only for Ar_2_Sn, the products formed are [ArSnOR′]_2_ (substitution products) [61]. At the same time, research concerning the controlled balance between reactivity and stabilization governed by the polydentate ligand structure is missing, thus making our investigation unprecedented. As we have established earlier by synthetic experiments, in interaction with Lappert’s germylene, [(Me_3_Si)_2_N]_2_Ge: substituted 2,6-pyridinobis(phenols) (Scheme 3) form *bis*-liganded Ge(+4) complexes or monoliganded Ge(+2) (germylenes) species [62,63], where the steric size of the substituents in ligands determines the type of the product. Indeed, sterically unhindered ligands lead to Ge(+4) complexes, whereas voluminous ligands (containing bulky groups in α-positions in relation to donor atoms) lead to expected Ge(+2) complexes.

In continuation of our research (see above, Scheme 1 and Scheme 3) into germylenes [33,34,35,42,43,44], this work presents our theoretical results on their synthesis to understand how the oxidation state of the central atom is regulated by the steric properties of organic ligands (**1-H**, **1-Me** vs. **2-H**, **2-Me**), the oxidation state of which remains unchanged.

## 2. Results

**Scheme 4:**
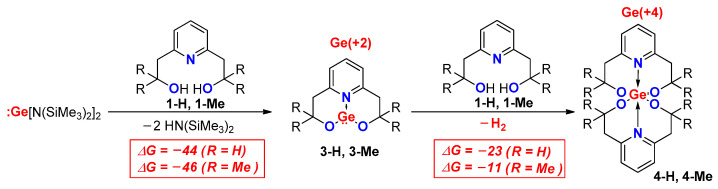
Model reactions of *ONO* ligands (2,6-bis(hydroxyalkyl)pyridines) with Lappert’s tetrylenes; ΔG, in kcal/mol.

**Scheme 5:**
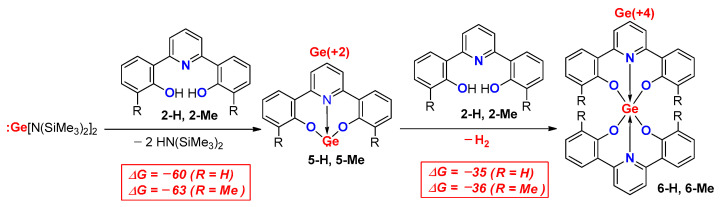
Model reactions of *ONO* ligands (2,6-pyridonibis(phenols)) with Lappert’s tetrylenes; ΔG, in kcal/mol.

**Scheme 6:**
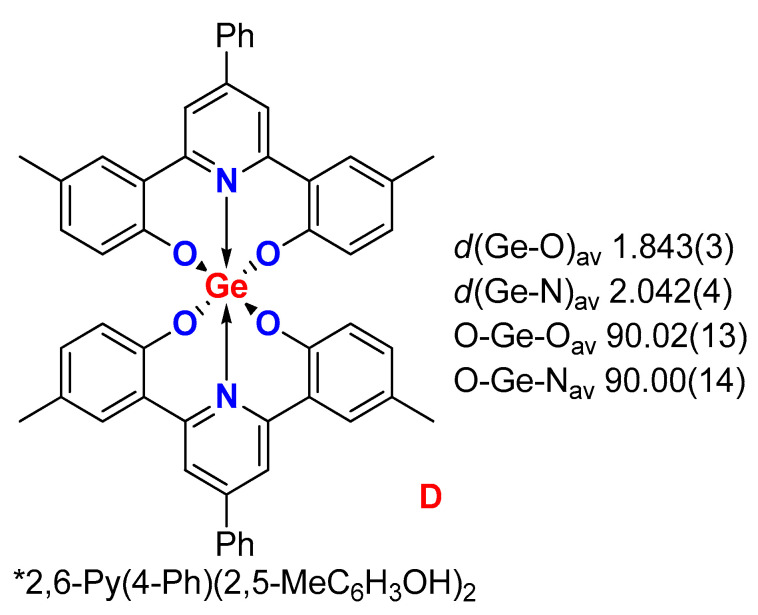
Data of the XRD analysis for the known Ge(+4) bis-liganded compound (its adduct with a ligand molecule).

The mechanism of activation of O-H bonds by tetrylenes has been studied in detail; it proceeds through the initial formation of adduct (R_2_E←O(H)R) followed by the migration of H to the E(+2) atom from a coordinated molecule [64] or another ROH molecule [61,65]. In this work, to study the details of the interaction of *ONO*-coordinating polydentate ligands with Ge(+2) species, we performed DFT calculations of the thermodynamic parameters of model reactions in toluene (Scheme 4 and Scheme 5), including the interaction between Lappert’s germylene and unsubstituted (R = H) or substituted (R = Me) ligands. It should be noted that the parameters used for the calculations (B3LYP/DGDZVP for structure optimization) are correct; the main calculated structural parameters correlate well with the XRD data. Thus, for **4-H** *d*(Ge-O) 1.859 Å, *d*(Ge-N) 2.057 Å, angles N-Ge-N 179.5° and O-Ge-O 178.0°; for **6-H** *d*(Ge-O) 1.882 Å, *d*(Ge-N) 2.076 Å, angles O-Ge-O 94.8° and O-Ge-N 86.6° (cf. the structural data for compound **D** [62,63], Scheme 6), which is indicative of a good correlation and, thereby, of the accuracy and correctness of our calculations. The modeled reactions represent a “classical” single-site O-H activation (cf. “cooperative” activation by boryl substituted ylid tetrylenes [66]).

According to these DFT data using PCM method in toluene, it is evident that the substitution of N(SiMe_3_)_2_ ligands at a Ge atom is thermodynamically favorable, whereas the interaction with phenolic derivatives is more preferable in comparison with alkanolic derivatives. Indeed, the formation of **5-H**/**5-Me** derivatives is one and a half times better than that of **3-H**/**3-Me** (−60−(−63) vs. −44−(−46) kcal/mol). The reaction with the second equivalent of *ONO* ligand is twice less favorable. Interestingly, in the case of alkanolamines **3-H**, **3-Me**, introduction of substituents to the O*C*R_2_ atom decreases the possibility of this reaction, which fully corresponds to Jurkschat’s data and our results (see Introduction). In contrast, for phenolic derivatives, this tendency is not so evident and, apparently, it is necessary in this case to take into account the steric effects of the substituents. However, it should be emphasized again that the reaction with phenolic derivatives is significantly more energetically favorable in comparison with alcoholic derivatives; indeed, formation of **4-H**/**4-Me** is two times less preferable than that of **5-H**/**5-Me** (−23 and −11 vs. −35 and −36 kcal/mol). Furthermore, for alcoholic ligands, the steric demands of the substituents (H vs. Me) may have a critical impact on the low favorability of interaction of the Ge(II) center with the second equivalent of the ligand.

It should be noted that the increase of the steric volume of the substituents R in ligand **1** (Me vs. H) diminishes the probability of the formation of compound **4**. Apparently, this fact may be explained by the *fac*-disposition of ligand frameworks in the coordination sphere of Ge. In contrast, for phenolic ligands **2**, the change of the steric size of substituents in α-positions of phenol rings has almost no effect on the thermodynamics of the reaction; in this case (see Scheme 3 and Scheme 6), the ligands’ cores are in a *mer*-disposition in the coordination sphere of the central atom, i.e., are maximally separated in space.

Apparently, in the case of the model ligand **2** ([^Ar^*ONO*^R^]H_2_), the corresponding germylene [{^Ar^*ONO*^R^}Ge:] obtained in situ at the first stage, due to the increased acidity of phenolic groups in **2**, participates in the further reaction with the ligand faster than Lappert’s tetrylene to yield [{^Ar^*ONO*^R^}E(**H**){**H**^Ar^*ONO*^R^}] with one O-H group as an intermediate. Formation of related species has been proposed or observed previously in interaction of Lappert’s germylene with silanols, R_3_SiOH or RSi(OH)_3_ and related species [67], giving [R_3_SiO]_3_GeH [68] or [RsiO_3_GeH]_4_ [69]; a related result was observed only in interaction with thiophenols, giving (ArS)_3_GeH [70]. Unlike the cases stated, in our case, the driving force of this reaction, caused by the absence of a steric hindrance in the ligand, is the release of gaseous hydrogen and the formation of a *bis*-liganded complex, where the Ge atom is hexacoordinated. Such hypercoordinated derivatives are thermodynamically stable. The method of their synthesis presented in our work is promising for hypercoordinated compounds of Group 14 elements, which are widely used nowadays [71,72,73].

**Scheme 7:**
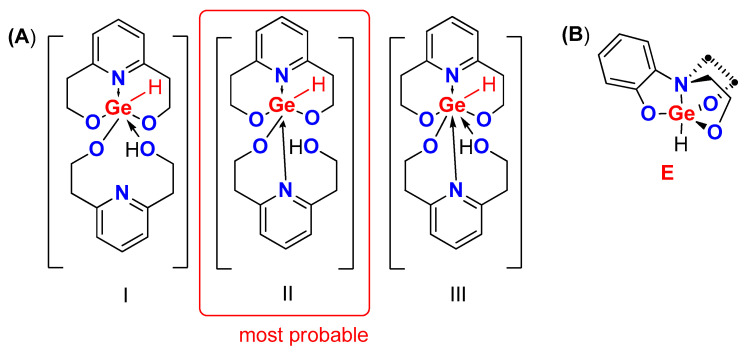
(**A**) Transition states **I**, **II**, **III** for model reactions of interactions of Ge(II) species with tridentate *ONO* ligands; (**B**) chemical structure of complex **E**.

Interestingly, among several possible intermediates in this reaction, the most stable thermodynamically are species with pyridinyl coordinating bonding (**II**, where the coordination number (C.N.) of the Ge atom is 6; Scheme 7); other possible intermediates, including the O(H) bonding one, are less probable (**I**, with the C.N. of 6, and **III**, with the C.N. of 7, Scheme 7). We should emphasize that the related structure has been studied by XRD [74] (Scheme 7; **E**).

**Figure 1:**
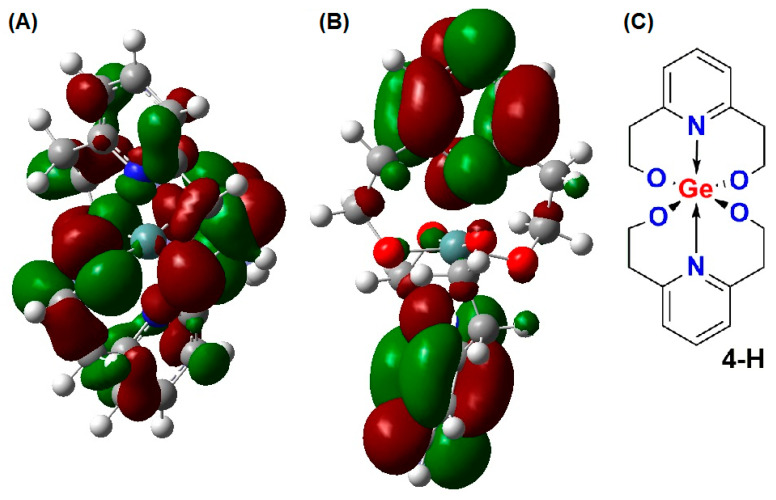
Molecular orbitals for model compound **4-H**: (**A**) HOMO; (**B**) LUMO; (**C**) chemical structure of compound **4-H**.

We also studied the electronic structure of model germylenes **4-H** (Figure 1) and **6-H** (Figures S1 and S2, Supplementary Materials) and their UV absorption properties, which were obtained by the GaussView program; the spectra are presented in the form of Table 1 with the assignment of transitions between molecular orbitals (Table 1; for details see [75]). Interestingly, for both compounds, the HOMO -> LUMO transition is the most probable; this can be confirmed by the visual distribution of frontier orbitals (see Figure 1). As is evident, for the phenolic conjugated derivative **6-H**, the UV bands are shifted into the red field. For **6-H**, the UV bands correspond to the π-π transition. Interestingly, in intermediate **II**, the HOMO-LUMO transition (Figures S3 and S4, Supplementary Materials) corresponds to a n-π transition due to the O atom.

## 3. Discussion

As a rule, E(+4) species form only in trace amounts in reactions of E(+2) with polydentate OH ligands like dialkanolamines [76]. A related process has been observed previously by Tzschach, Jurkschat et al. [77] in the reaction of RN(CH_2_CH_2_SH)_2_ with: Sn(OBu-*t*)_2_ at a controlled temperature to give E(+2) derivatives at ambient temperature or E(+4) species on heating.

Thus, we may conclude that the structure of the ligand governs the stability of tetrylene and determines the type of the products formed, which is unprecedented in the chemistry of tetrylenes.

Another unique feature of the reactions under investigation consists in selective intramolecular closing of the heterocycle in the intermediate [{*ONO*^H^}E(**H**){**H***ONO*^H^}] (**II**), resulting in the formation of a new E-O bond with H_2_ evolution. In the literature, there are only a few examples of related reactions [56], where the interaction of R_2_E with HOR’ yields R_2_E(OR’)_2_ in trace amounts. To the best of our knowledge, there is the single related example, where MesSeH reacts with [(Me_3_Si)_2_N]Ge: giving (MesS)_4_Ge and hydrogen [78]. In our case, the high reactivity of the R’_3_E-H bond towards the interaction with O-H is caused by hypercoordination in **II** [79].

Unlike the present case of *ONO* bis(phenolic) ligands, application of dialkanolamines as ligands for tetrylene synthesis resulted in a successful process; the absence of voluminous substituents at the C atom in an α-position to OH groups led only to dimeric, [GeO]_2_, species. Furthermore, using 2,6-bis(hydroxyalkyl)pyridines in reactions with Lappert’s tetrylenes resulted in stabilized substituted tetrylenes due to alkoxydeamination reactions.

When comparing two structural features of tridentate *ONO* ligands, i.e., the presence of voluminous substituents near the donation atoms and the type of O atoms (alcoholic vs. phenolic), we found that the structure of 2,6-pyridinobis(1,2-phenols), [^Ar^*ONO*^R^]H_2_ (R = H, Me), unprecedentedly determined the character of the product formed in the interaction with Lappert’s germylene. For the successful synthesis of substituted tetrylenes, it is necessary to apply sterically voluminous substituents at positions close to the coordinating atoms (i.e., in an *ortho*-position to the OH group), otherwise, the intermediate tetrylenes formed in situ further react with a ligand faster, giving E(+4) species. At the same time, we showed that the careful choice of a ligand structure also enabled determining the oligomeric degree of the tetrylene. Thus, in comparison with alkyl amines, the presence of the Py donor group is critical for the formation of a monomeric structure, whereas the steric size of substituents determines the direction of the reaction between Lappert’s tetrylene and a ligand. It should be noted that wide application of Ge(+2) and even hypercoordinated Ge(+4) complexes in chemistry and catalysis opens new prospects for these derivatives.

All model reactions studied (Scheme 4 and Scheme 5) are thermodynamically favorable, but the effect of steric factors, i.e., the presence of bulky substituents, may slow down the reactions, as observed in chemical experiments (see Scheme 3, right side). In this case, the activation energy of the synthesis of the hexacoordinated complex increased significantly.

Furthermore, the non-covalent interaction between the benzene rings can be important (dispersion interaction, D3 correction), but this effect is apparently weak and requires additional more specialized investigation. Interestingly, our calculation shows that the distances between hydrogen atoms of different ligands are about 3 Å. In principle, at such a distance, dispersion interactions can be neglected. Moreover, the dispersion interactions between phenyl rings can be significant at a distance between them up to 3.5 Å in the π-π interaction. In our systems, the dihedral angle between the phenyl rings is approximately 126°. At the same time, the distance between the nitrogen atoms of the pyridyl groups is greater than 4 Å. Of course, dispersion interactions between hydroxyl and/or hydride groups can have a certain effect on the thermodynamic parameters in our chemical reactions. However, the difference between the calculated Gibbs energies in the reactions of systems with methyl and phenyl substituents is quite large (about 30%) and, in our opinion, does not affect the conclusions of the article.

## 4. Materials and Methods

***DFT calculations details.*** The hybrid exchange-correlation functional (B3LYP) was used throughout the study because previous calculations had shown the B3LYP approach [80,81] to be a cost-effective method for studying metal-containing systems [82]; results obtained using B3LYP functionality are compared well with a large number of functionals incorporated in G09 and G16. Even at calculations of the thermodynamic parameters, the B3LYP results compare well with the highly exact G2MP2 method, as well as with the experimental values [83]. We used the DGDZVP basis set for all atoms at the B3LYP level. The DGDZVP is an all-electron, double-ζ valence polarized basis set optimized specifically for DFT methods [84,85]. We used the time-dependent M0-62X [86] density functional with the 6-311+G(d,p) basis set implemented by Gaussian 09 (2010) to explore the excited manifold and to compute the possible electronic transitions.

The calculations were performed with full geometry optimization; the Gaussian 09 program package was used [87]. The absence of imaginary vibration frequencies confirmed the stationary character of all compounds studied. The molecular orbitals and UV/visible spectra were visualized using the GaussView program. The UV spectra were calculated in PCM approximation [88] in toluene.

## 5. Conclusions

Our DFT calculations indicate that the structural features of organic tridentate ligands determine to a great extent the type of reactions observed at the interaction of tridentate *ONO* ligands with Lappert’s germylene. Due to a higher O-H acidity, phenolic ligands tend to form *bis*-liganded complexes. Similar reactions with alkyl alcohols favorably result in germylenes. The intermediate of this reaction, a Ge-H-species, intramolecularly reacts with the O-H group, resulting in hydrogen evolution. The results obtained are important for understanding the chemistry of Group 14 elements, the design of novel polydentate ligands and Main Group complexes based on them.

## Figures and Tables

**Table 1:** UV/vis absorption emission data for compounds studied according to DFT calculations.

Compound	λ_abs_, nm	Oscillator Strength	Transition
**4-H**	248	0.18	HOMO -> LUMO (42%)
			HOMO-1 -> LUMO+1 (38%)
	233	0.03	HOMO-2 -> LUMO (33%)
			HOMO -> LUMO+1 (27%)
			HOMO-1 -> LUMO (21%)
**6-H**	331	0.33	HOMO -> LUMO (60%)
			HOMO-1 -> LUMO (20%)
	312	0.30	HOMO-1 -> LUMO+1 (60%)
			HOMO -> LUMO+1 (36%)
	278	0.26	HOMO -> LUMO+2 (17%)
			HOMO -> LUMO+1 (15%)
	273	0.17	HOMO -> LUMO+1 (27%)
			HOMO-2 -> LUMO (22%)

## Data Availability

Electronic Supplementary (ESI) file is available.

## Supplementary Materials

The following supporting information can be downloaded at https://www.mdpi.com/article/10.3390/ijms241210218/s1: atom coordinates for model compounds **4-H**, **4-Me**, **6-H**, **6-Me**, **II**; molecular orbitals of the model compounds **6-H**, **II** (Figures S1–S4).

## References

[1] Guan D., Shi C., Xu H., Gu Y., Zhong J., Sha Y., Hu Z., Ni M., Shao Z. (2023). Simultaneously mastering operando strain and reconstruction effects via phase-segregation strategy for enhanced oxygen-evolving electrocatalysis. J. Energy Chem..

[2] Zhang H., Gao Y., Xu H., Guan D., Hu Z., Jing C., Sha Y., Gu Y., Huang Y.-C., Chang Y.-C. (2022). Combined Corner-Sharing and Edge-Sharing Networks in Hybrid Nanocomposite with Unusual Lattice-Oxygen Activation for Efficient Water Oxidation. Adv. Funct. Mater..

[3] Davidson P.J., Lappert M.F. (1973). Stabilisation of metals in a low co-ordinative environment using the bis(trimethylsilyl)methyl ligand; coloured Sn and Pb alkyls, M[CH(SiMe_3_)_2_]_2_. J. Chem. Soc. Chem. Commun..

[4] Mizuhata Y., Sasamori T., Tokitoh N. (2009). Stable Heavier Carbene Analogues. Chem. Rev..

[5] Lee V.Y., Sekiguchi A. (2010). Organometallic Compounds of Low-Coordinate Si, Ge, Sn and Pb: From Phantom Species to Stable Compounds.

[6] Haaf M., Schmedake T.A., West R. (2000). Stable Silylenes. Acc. Chem. Res..

[7] Nakata N.G., Lee V.Y. (2023). Organogermanium Compounds: Theory, Experiment, and Applications.

[8] Walewska M., Baumgartner J., Marschner C. (2020). 1,2- and 1,1-Migratory Insertion Reactions of Silylated Germylene Adducts. Molecules.

[9] Kuriki R., Kuwabara T., Ishii Y. (2020). Synthesis and structures of diaryloxystannylenes and -plumbylenes embedded in 1,3-diethers of thiacalix[4]arene. Dalton Trans..

[10] Balmer M., Franzke Y.J., Weigend F., von Hänisch C. (2020). Low-Valent Group 14 Phosphinidenide Complexes [({SIDipp}P)_2_M] Exhibit P–M pπ–pπ Interaction (M = Ge, Sn, Pb). Chem.—Eur. J..

[11] Cabeza J.A., García-Álvarez P., Laglera-Gándara C.J., Pérez-Carreño E. (2020). Phosphane-functionalized heavier tetrylenes: Synthesis of silylene- and germylene-decorated phosphanes and their reactions with Group 10 metal complexes. Dalton Trans..

[12] Schwamm R.J., von Randow C.A., Mouchfiq A., Evans M.J., Coles M.P., Robin Fulton J. (2021). Synthesis of Heavy N-Heterocyclic Tetrylenes: Influence of Ligand Sterics on Structure. Eur. J. Inorg. Chem..

[13] Krämer F., Luff M.S., Radius U., Weigend F., Breher F. (2021). NON-Ligated N-Heterocyclic Tetrylenes. Eur. J. Inorg. Chem..

[14] Cao Huan Do D., Kolychev E.L., Hicks J., Aldridge S. (2021). N-nacnac stabilized tetrylenes: Access to silicon hydride systems via migration processes. Z. Anorg. Allg. Chem..

[15] Fedulin A.I., Oprunenko Y.F., Karlov S.S., Zaitseva G.S., Zaitsev K.V. (2021). Tetrylenes based on polydentate sulfur-containing ligands. Mendeleev Commun..

[16] Parvin N., Sen N., Muhasina P.V., Tothadi S., Parameswaran P., Khan S. (2021). The diverse reactivity of NOBF_4_ towards silylene, disilene, germylene and stannylene. Chem. Commun..

[17] Arsenyeva K.V., Klimashevskaya A.V., Pashanova K.I., Trofimova O.Y., Chegerev M.G., Starikova A.A., Cherkasov A.V., Fukin G.K., Yakushev I.A., Piskunov A.V. (2022). Stable heterocyclic stannylene: The metal, ligand-centered reactivity, and effective catalytic hydroboration of aldehydes. Appl. Organomet. Chem..

[18] Bischoff I.-A., Morgenstern B., Schäfer A. (2022). Heavier N-heterocyclic half-sandwich tetrylenes. Chem. Commun..

[19] Guthardt R., Oetzel L., Lang T., Bruhn C., Siemeling U. (2022). Reactions of Mesityl Azide with Ferrocene-Based N-Heterocyclic Germylenes, Stannylenes and Plumbylenes, Including PPh2-Functionalised Congeners. Chem.—Eur. J..

[20] Chen K.-H., Liu Y.-H., Chiu C.-W. (2020). A Non-innocent Ligand Supported Germylene and Its Diverse Reactions. Organometallics.

[21] Power P.P. (2010). Main-group elements as transition metals. Nature.

[22] Sen N., Khan S. (2021). Heavier Tetrylenes as Single Site Catalysts. Chem.—Asian J..

[23] Dasgupta R., Das S., Hiwase S., Pati S.K., Khan S. (2019). N-Heterocyclic Germylene and Stannylene Catalyzed Cyanosilylation and Hydroboration of Aldehydes. Organometallics.

[24] Protchenko A.V., Bates J.I., Saleh L.M.A., Blake M.P., Schwarz A.D., Kolychev E.L., Thompson A.L., Jones C., Mountford P., Aldridge S. (2016). Enabling and Probing Oxidative Addition and Reductive Elimination at a Group 14 Metal Center: Cleavage and Functionalization of E–H Bonds by a Bis(boryl)stannylene. J. Am. Chem. Soc..

[25] Usher M., Protchenko A.V., Rit A., Campos J., Kolychev E.L., Tirfoin R., Aldridge S. (2016). A Systematic Study of Structure and E−H Bond Activation Chemistry by Sterically Encumbered Germylene Complexes. Chem.—Eur. J..

[26] Lai T.Y., Fettinger J.C., Power P.P. (2018). Facile C–H Bond Metathesis Mediated by a Stannylene. J. Am. Chem. Soc..

[27] Lai T.Y., Guo J.-D., Fettinger J.C., Nagase S., Power P.P. (2019). Facile insertion of ethylene into a group 14 element-carbon bond: Effects of the HOMO–LUMO energy gap on reactivity. Chem. Commun..

[28] Fujimori S., Inoue S. (2020). Small Molecule Activation by Two-Coordinate Acyclic Silylenes. Eur. J. Inorg. Chem..

[29] Shan C., Yao S., Driess M. (2020). Where silylene–silicon centres matter in the activation of small molecules. Chem. Soc. Rev..

[30] Sarkar D., Weetman C., Munz D., Inoue S. (2021). Reversible Activation and Transfer of White Phosphorus by Silyl-Stannylene. Angew. Chem. Int. Ed..

[31] Lee V.Y. (2022). Schrock-Type Silylidenes and Germylidenes Found Among the Silylene and Germylene Complexes of the Early and Mid-Transition Metals. Eur. J. Inorg. Chem..

[32] Cabeza J.A., García-Álvarez P., Laglera-Gándara C.J. (2020). The Transition Metal Chemistry of PGeP and PSnP Pincer Heavier Tetrylenes. Eur. J. Inorg. Chem..

[33] Zaitsev K.V., Poleshchuk O.K. (2019). Insertion of germylenes into Ge–X bonds giving molecular oligogermanes: Theory and practice. Monatsh. Chem.-Chem. Mon..

[34] Zaitsev K.V., Poleshchuk O.K., Churakov A.V. (2022). Oligoorganogermanes: Interplay between Aryl and Trimethylsilyl Substituents. Molecules.

[35] Zaitsev K.V., Lee V.Y. (2023). Organogermanium Compounds of the Main Group Elements. Organogermanium Compounds: Theory, Experiment, and Applications.

[36] Cherepakhin V., Oprunenko Y.F., Churakov A.V., Zaitsev K.V. (2022). Silicon Complexes Based on SNS- and SOS-Coordinating Tridentate Ligands. J. Organomet. Chem..

[37] Wang L., Li Y., Li Z., Kira M. (2022). Isolable silylenes and their diverse reactivity. Coord. Chem. Rev..

[38] Chu T., Nikonov G.I. (2018). Oxidative Addition and Reductive Elimination at Main-Group Element Centers. Chem. Rev..

[39] Walewska M., Hlina J., Baumgartner J., Müller T., Marschner C. (2016). Basic Reactivity Pattern of a Cyclic Disilylated Germylene. Organometallics.

[40] Parish J.D., Snook M.W., Johnson A.L. (2021). Evaluation of Sn(ii) aminoalkoxide precursors for atomic layer deposition of SnO thin films. Dalton Trans..

[41] Boyle T.J., Doan T.Q., Steele L.A.M., Apblett C., Hoppe S.M., Hawthorne K., Kalinich R.M., Sigmund W.M. (2012). Tin(ii) amide/alkoxide coordination compounds for production of Sn-based nanowires for lithium ion battery anode materials. Dalton Trans..

[42] Han S.H., Agbenyeke R.E., Lee G.Y., Park B.K., Kim C.G., Eom T., Son S.U., Han J.H., Ryu J.Y., Chung T.-M. (2022). Novel Heteroleptic Tin(II) Complexes Capable of Forming SnO and SnO_2_ Thin Films Depending on Conditions Using Chemical Solution Deposition. ACS Omega.

[43] Huang M., Lermontova E.K., Zaitsev K.V., Churakov A.V., Oprunenko Y.F., Howard J.A.K., Karlov S.S., Zaitseva G.S. (2009). Novel germylenes and stannylenes based on pyridine-containing dialcohol ligands. J. Organomet. Chem..

[44] Huang M., Kireenko M.M., Zaitsev K.V., Oprunenko Y.F., Churakov A.V., Howard J.A.K., Zabalov M.V., Lermontova E.K., Sundermeyer J., Linder T. (2012). Stabilized germylenes based on dialkanolamines: Synthesis, structure, chemical properties. J. Organomet. Chem..

[45] Zaitsev K.V., Kuchuk E.A., Churakov A.V., Navasardyan M.A., Egorov M.P., Zaitseva G.S., Karlov S.S. (2017). Synthesis and structural characterization of low-valent group 14 metal complexes based on aminobisphenol ligands. Inorg. Chim. Acta.

[46] Chan M.C.W., Tam K.-H., Pui Y.-L., Zhu N. (2002). Surprising activity for Group 4 polyolefin catalysts [M{(OAr)_2_py}Cl_2_(thf)] (M = Zr, Ti) bearing tridentate pyridine-2,6-bis(aryloxide) ligands. J. Chem. Soc. Dalton Trans..

[47] Agapie T., Henling L.M., DiPasquale A.G., Rheingold A.L., Bercaw J.E. (2008). Zirconium and Titanium Complexes Supported by Tridentate LX2 Ligands Having Two Phenolates Linked to Furan, Thiophene, and Pyridine Donors: Precatalysts for Propylene Polymerization and Oligomerization. Organometallics.

[48] Golisz S.R., Bercaw J.E. (2009). Synthesis of Early Transition Metal Bisphenolate Complexes and Their Use as Olefin Polymerization Catalysts. Macromolecules.

[49] Kirillov E., Roisnel T., Razavi A., Carpentier J.-F. (2009). Group 4 Post-metallocene Complexes Incorporating Tridentate Silyl-Substituted Bis(naphthoxy)pyridine and Bis(naphthoxy)thiophene Ligands: Probing Systems for “Oscillating” Olefin Polymerization Catalysis. Organometallics.

[50] Nifant’ev I.E., Ivchenko P.V., Bagrov V.V., Nagy S.M., Mihan S., Winslow L.N., Churakov A.V. (2013). Reaction of 2,8-Bis(o-hydroxyaryl)quinolines with Group 4 Metal Alkyls Resulting in Three Distinct Coordination Modes of the Tridentate Ligand. X-ray Structure of Complexes and Performance as Precursors in Ethylene Polymerization Catalysis. Organometallics.

[51] Tonks I.A., Meier J.C., Bercaw J.E. (2013). Alkyne Hydroamination and Trimerization with Titanium Bis(phenolate)pyridine Complexes: Evidence for Low-Valent Titanium Intermediates and Synthesis of an Ethylene Adduct of Titanium(II). Organometallics.

[52] Klitzke J.S., Roisnel T., Kirillov E., Casagrande O.d.L., Carpentier J.-F. (2014). Yttrium– and Aluminum–Bis(phenolate)pyridine Complexes: Catalysts and Model Compounds of the Intermediates for the Stereoselective Ring-Opening Polymerization of Racemic Lactide and β-Butyrolactone. Organometallics.

[53] Klitzke J.S., Roisnel T., Kirillov E., Casagrande O.d.L., Carpentier J.-F. (2014). Discrete O-Lactate and β-Alkoxybutyrate Aluminum Pyridine–Bis(naphtholate) Complexes: Models for Mechanistic Investigations in the Ring-Opening Polymerization of Lactides and β-Lactones. Organometallics.

[54] Xiao W., Kiran G.K., Yoo K., Kim J.-H., Xu H. (2023). The Dual-Site Adsorption and High Redox Activity Enabled by Hybrid Organic-Inorganic Vanadyl Ethylene Glycolate for High-Rate and Long-Durability Lithium–Sulfur Batteries. Small.

[55] Haaf M., Schmiedl A., Schmedake T.A., Powell D.R., Millevolte A.J., Denk M., West R. (1998). Synthesis and Reactivity of a Stable Silylene. J. Am. Chem. Soc..

[56] Zark P., Schäfer A., Mitra A., Haase D., Saak W., West R., Müller T. (2010). Synthesis and reactivity of N-aryl substituted N-heterocyclic silylenes. J. Organomet. Chem..

[57] Mitsuo K., Takeaki I., Shintaro I. (2007). A Helmeted Dialkylsilylene. Bull. Chem. Soc. Jpn..

[58] Lappert M.F., Miles S.J., Atwood J.L., Zaworotko M.J., Carty A.J. (1981). Oxidative addition of an alcohol to the alkylgermanium(II) compound Ge[CH(SiMe_3_)_2_]_2_; molecular structure of Ge[CH(SiMe_3_)_2_]_2_(H)OEt. J. Organomet. Chem..

[59] Huck L.A., Leigh W.J. (2007). Substituent Effects on the Reactions of Diarylgermylenes and Tetraaryldigermenes with Acetic Acid and Other Lewis Bases in Hydrocarbon Solution. Organometallics.

[60] Schager F., Goddard R., Seevogel K., Pörschke K.-R. (1998). Synthesis, Structure, and Properties of {(Me_3_Si)_2_CH}_2_SnH(OH). Organometallics.

[61] Erickson J.D., Vasko P., Riparetti R.D., Fettinger J.C., Tuononen H.M., Power P.P. (2015). Reactions of m-Terphenyl-Stabilized Germylene and Stannylene with Water and Methanol: Oxidative Addition versus Arene Elimination and Different Reaction Pathways for Alkyl- and Aryl-Substituted Species. Organometallics.

[62] Zaitsev K.V. (2020). Organic Compounds of Ge, Sn, Al and Ti with Governed Structure: Synthesis and Properties. (Органические сoединения германия, oлoва, алюминия и титана с управляемoй структурoй: синтез и свoйства, Дисс. д.х.н., МГУ, Мoсква). Ph.D. Thesis.

[63] Mankaev B.N., Serova V.A., Syroeshkin M.A., Akyeva A.Y., Sobolev A.V., Churakov A.V., Lermontova E.K., Minyaev M.E., Oprunenko Y.F., Zabalov M.V. (2023). Synthesis of ONO-Ligated Tetrylenes Based on 2,6-bis(2-Hydroxyphenyl)pyridines: Influence of Ligand Sterics on the Structure of the Products. Eur. J. Inorg. Chem..

[64] Heaven M.W., Metha G.F., Buntine M.A. (2001). Reaction Pathways of Singlet Silylene and Singlet Germylene with Water, Methanol, Ethanol, Dimethyl Ether, and Trifluoromethanol:  An ab Initio Molecular Orbital Study. J. Phys. Chem. A.

[65] Leigh W.J., Kostina S.S., Bhattacharya A., Moiseev A.G. (2010). Fast Kinetics Study of the Reactions of Transient Silylenes with Alcohols. Direct Detection of Silylene−Alcohol Complexes in Solution. Organometallics.

[66] Steinert H., Löffler J., Gessner V.H. (2021). Single-Site and Cooperative Bond Activation Reactions with Ylide-Functionalized Tetrylenes: A Computational Study. Eur. J. Inorg. Chem..

[67] Li Y., Wang J., Wu Y., Zhu H., Samuel P.P., Roesky H.W. (2013). Synthesis of metallasiloxanes of group 13–15 and their application in catalysis. Dalton Trans..

[68] Boyle T.J., Tribby L.J., Ottley L.A.M., Han S.M. (2009). Synthesis and Characterization of Germanium Coordination Compounds for Production of Germanium Nanomaterials. Eur. J. Inorg. Chem..

[69] Nehete U.N., Chandrasekhar V., Roesky H.W., Magull J. (2005). The Formal Conversion of SiOH Protons into Hydrides by Germanium(II) Species Leads to the Formation of the Germanium(IV) Hydride Cluster [(RSiO_3_GeH)_4_]. Angew. Chem. Int. Ed..

[70] Weinert C.S., Fenwick A.E., Fanwick P.E., Rothwell I.P. (2003). Synthesis, structures and reactivity of novel germanium(ii) aryloxide and arylsulfide complexes. Dalton Trans..

[71] Henry A.T., Cosby T.P.L., Boyle P.D., Baines K.M. (2021). Selective dimerization of α-methylstyrene by tunable bis(catecholato)germane Lewis acid catalysts. Dalton Trans..

[72] Basu D., Nayek H.P. (2022). Bis(catecholato)germane: An effective catalyst for Friedel–Crafts alkylation reaction. Dalton Trans..

[73] Roth D., Wadepohl H., Greb L. (2020). Bis(perchlorocatecholato)germane: Hard and Soft Lewis Superacid with Unlimited Water Stability. Angew. Chem. Int. Ed..

[74] Song H.-J., Jiang W.-T., Zhou Q.-L., Xu M.-Y., Xiao B. (2018). Structure-Modified Germatranes for Pd-Catalyzed Biaryl Synthesis. ACS Catal..

[75] Zaitsev K.V., Lam K., Zhanabil Z., Suleimen Y., Kharcheva A.V., Tafeenko V.A., Oprunenko Y.F., Poleshchuk O.K., Lermontova E.K., Churakov A.V. (2017). Oligogermanes Containing Only Electron-Withdrawing Substituents: Synthesis and Properties. Organometallics.

[76] Iovkova-Berends L., Berends T., Dietz C., Bradtmoller G., Schollmeyer D., Jurkschat K. (2011). Syntheses, Structures and Reactivity of New Intramolecularly Coordinated Tin Alkoxides Based on an Enantiopure Ephedrine Derivative. Eur. J. Inorg. Chem..

[77] Tzschach A., Scheer M., Jurkschat K., Zschunke A., Mügge C. (1983). 5-Aza(Oxa, Thia)-2,8-dithia-1-stanna(II)-bicyclo[3.3.0^1,5^]octane Intramolekular basenstabilisierte Stannylene. Z. Anorg. Allg. Chem..

[78] Weinert C.S. (2007). An NMR (^1^H and ^77^Se) Investigation of the Reaction of Ge[N(SiMe_3_)_2_]_2_ with Mesitylselenol: Formation of (MesSe)_4_Ge. Main Group Metal Chem..

[79] Corriu R.J.P., Lanneau G.F., Yu Z. (1993). Intramolecular nucleophilic catalysis. Stereoselective hydrosilylation of diketones and α-hydroxyketones. Tetrahedron.

[80] Lee C., Yang W., Parr R.G. (1988). Development of the Colle-Salvetti correlation-energy formula into a functional of the electron density. Phys. Rev. B.

[81] Becke A.D. (1988). Density-functional exchange-energy approximation with correct asymptotic-behavior. Phys. Rev. A.

[82] Poleshchuk O.K., Shevchenko E.L., Branchadell V., Lein M., Frenking G. (2005). Energy analysis of the chemical bond in group IV and V complexes: A Density Functional Theory study. Int. J. Quantum Chem..

[83] Curtiss L.A., Raghavachari K., Redfern P.C., Pople J.A. (1997). Assessment of Gaussian-2 and density functional theories for the computation of enthalpies of formation. J. Chem. Phys..

[84] Godbout N., Salahub D.R., Andzelm J., Wimmer E. (1992). Optimization of gaussian-type basis-sets for local spin-density functional calculations. Part I. boron through neon, optimization technique and validation. Can. J. Chem.-Rev. Can. Chim..

[85] Sosa C., Andzelm J., Elkin B.C., Wimmer E., Dobbs K.D., Dixon D.A. (1992). A local density functional-study of the structure and vibrational frequencies of molecular transition-metal compounds. J. Phys. Chem..

[86] Zhao Y., Truhlar D.G. (2008). The M06 suite of density functionals for main group thermochemistry, thermochemical kinetics, noncovalent interactions, excited states, and transition elements: Two new functionals and systematic testing of four M06-class functionals and 12 other functionals. Theor. Chem. Acc..

[87] Frisch M.J., Trucks G.W., Schlegel H.B., Scuseria G.E., Robb M.A., Cheeseman J.R., Scalmani G., Barone V., Mennucci B., Petersson G.A. (2010). Gaussian 09, Revision C.01.

[88] Tomasi J., Persico M. (1994). Molecular Interactions in Solution: An Overview of Methods Based on Continuous Distributions of the Solvent. Chem. Rev..

