# Supplementary material for: Germanium Complexes with ONO Tridentate Ligands: O-H Bond Activation Control According to DFT Calculations
Source: Int J Mol Sci. 2023 Jun 16;24(12):10218. doi: 10.3390/ijms241210218 (PMC10299505; doi:10.3390/ijms241210218)
Supplement: Supplementary file 1 [file ijms-24-10218-s001.zip › ijms-2418355-supplementary.pdf]

SUPPORTING INFORMATION

for

Germanium Complexes with *O**N**O* Tridentate Ligands: O-H Bond Activation Control According to DFT Calculations

Kirill V. Zaitsev [1], Andrey D. Trubachev [1] and Oleg Kh. Poleshchuk [2,3,]*

[1] Chemistry Department, M.V. Lomonosov Moscow State University, Leninskye Gory 1, 3, Moscow 119991, Russia
[2] National Research Tomsk State University, Lenin Av., 36, Tomsk 634050, Russia
[3] Tomsk State Pedagogical University, Kievskaya Str., 60, Tomsk 634061, Russia
***** Correspondence: poleshch@tspu.edu.ru

Table of Content

Atom coordinates for model compounds

4-H……………………………………………………………………………………………..S3

4-Me………………………………………………………………………..………………..S6

6-H………………………………………………………………………...…………………..S9

6-Me…………………………………………………………………………………………..S12

II…………………………………..……………………………………………………..S16

Molecular orbitals of the model compounds

Figure S1. HOMO orbital of compound **6-H**……..………………………………………………..S19

Figure S2. LUMO orbital of compound **6-H**……..………………………………………………..S20

Figure S3. HOMO orbital of compound **II**………....……………………………………………..S21

Figure S4. LUMO orbital of compound **II**……….....…………………………………………..S22

Atom coordinates for model compounds

4-H

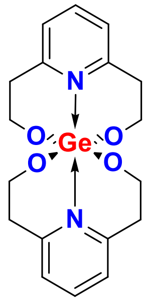

G= - 3184.330 a.e.

Ge	0.00000000	0.00001000	-0.08125000
O	-0.34313500	-1.26885000	1.25539900
O	-0.13654100	1.32036500	-1.36714600
O	0.34314000	1.26881800	1.25545300
O	0.13652000	-1.32026200	-1.36723000
C	0.71569900	-1.57272500	2.11556000
C	1.96626200	-2.05867800	1.35659400
C	0.87341600	2.28574700	-1.43061200
C	2.20803300	1.66921500	-1.87035100
N	2.16086200	-0.09549500	-0.12962300
N	-2.16086300	0.09548700	-0.12964700
C	2.88752400	0.79434200	-0.84579100
C	2.78068500	-0.98307000	0.68497600
C	4.27638100	0.85746000	-0.70071000
C	4.16604400	-0.95058200	0.85424600
C	4.92398400	-0.00920100	0.16906500
C	-2.78068500	0.98308000	0.68493200

C	-2.88752400	-0.79438600	-0.84577200
C	-4.16604100	0.95058500	0.85422200
C	-4.27637800	-0.85751600	-0.70066500
C	-4.92397900	0.00916900	0.16908700
H	0.39217700	-2.38436000	2.78473500
H	0.99289500	-0.71025300	2.74231700
H	1.62287400	-2.75370800	0.57983400
H	2.62550700	-2.61201100	2.03547600
H	1.00802800	2.79635000	-0.46652600
H	0.58390200	3.03774100	-2.18014000
H	2.91187900	2.45411900	-2.17298200
H	1.99992300	1.05222800	-2.75541900
H	4.83418100	1.58623600	-1.27921600
H	4.63463600	-1.66817200	1.51937900
H	6.00226800	0.03483200	0.29659600
H	-4.63463200	1.66819400	1.51933700
H	-4.83417900	-1.58632000	-1.27913500
H	-6.00226100	-0.03487300	0.29663500
C	-0.87337000	-2.28571800	-1.43065600
C	-2.20804900	-1.66928000	-1.87032900
C	-0.71573100	1.57274100	2.11555100
C	-1.96625300	2.05870100	1.35651400
H	-1.00790300	-2.79633500	-0.46656800
H	-0.58383800	-3.03768700	-2.18020200
H	-2.91187400	-2.45423800	-2.17287300
H	-2.00004100	-1.05232800	-2.75544600

H	-0.39222500	2.38438700	2.78472100
H	-0.99297600	0.71029300	2.74232100
H	-1.62281200	2.75367200	0.57972700
H	-2.62550100	2.61209100	2.03534600

4-Me

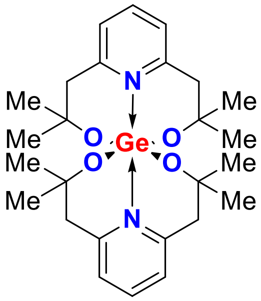

G= - 3498.638 a.e.

Ge	-0.00001800	-0.00002800	-0.08948000
O	0.32467300	1.27174700	1.26986200
O	0.09729600	-1.35183300	-1.38115200
O	-0.32426500	-1.27257700	1.26936100
O	-0.09769200	1.35230400	-1.38060800
C	-0.69543700	1.93197000	2.00120500
C	-1.88281100	2.29783700	1.07591800
C	-0.96068800	-2.13743400	-1.88091500
C	-2.19529700	-1.25507800	-2.16545200
N	-2.11681700	0.23484300	-0.21715900
N	2.11680200	-0.23471100	-0.21787200
C	-2.85999600	-0.59103400	-0.98924700
C	-2.71849300	1.15469400	0.57137500
C	-4.24315000	-0.66410800	-0.81459700
C	-4.10046200	1.12009200	0.77084200
C	-4.86661000	0.16712800	0.10989600
C	2.71872600	-1.15484200	0.57014400
C	2.85974300	0.59178900	-0.98952300
C	4.10069000	-1.12001200	0.76960100

C	4.24288600	0.66507100	-0.81490500
C	4.86657700	-0.16651500	0.10912800
H	-1.46738800	2.78570300	0.18745200
H	-2.54259800	3.00522400	1.58956500
H	-2.95190000	-1.83214200	-2.70861800
H	-1.85408300	-0.43992100	-2.81670500
H	-4.81803200	-1.35048300	-1.42714300
H	-4.55904700	1.85421900	1.42464700
H	-5.94055000	0.11348600	0.26676900
H	4.55945300	-1.85437800	1.42301400
H	4.81760600	1.35188800	-1.42710700
H	5.94050900	-0.11268200	0.26598700
C	0.96008900	2.13838900	-1.88009200
C	2.19479000	1.25637600	-2.16528900
C	0.69607400	-1.93330700	1.99989200
C	1.88325000	-2.29840300	1.07407700
H	2.95125100	1.83382600	-2.70824800
H	1.85360700	0.44153400	-2.81695200
H	1.46766000	-2.78567000	0.18535700
H	2.54320700	-3.00610900	1.58707400
C	1.34043000	3.28471200	-0.92464400
H	2.14883400	3.89764700	-1.34451100
H	0.47316200	3.93549200	-0.76669000
H	1.64722700	2.89317700	0.04690000
C	0.46355500	2.72110900	-3.21455100
H	1.20694800	3.38417800	-3.67400000

H	0.22646500	1.91565900	-3.91820700
H	-0.45178500	3.29634800	-3.03939600
C	-0.08126500	3.23246100	2.54706700
H	0.19779600	3.91037100	1.73473900
H	-0.77660700	3.75491700	3.21575200
H	0.82442200	2.99107300	3.11351800
C	-1.23331300	1.09328200	3.17764300
H	-0.46543600	0.97943900	3.94695900
H	-2.09819800	1.58721900	3.64049100
H	-1.52363900	0.09903300	2.83189200
C	-0.46450400	-2.71957600	-3.21576500
H	-1.20804400	-3.38241600	-3.67530900
H	-0.22757400	-1.91382300	-3.91912500
H	0.45085100	-3.29494000	-3.04110400
C	-1.34106800	-3.28416800	-0.92596800
H	-0.47380500	-3.93502700	-0.76828700
H	-1.64783400	-2.89300400	0.04573500
H	-2.14948400	-3.89690200	-1.34609500
C	0.08213500	-3.23422600	2.54498600
H	-0.19711900	-3.91159700	1.73227300
H	0.77769300	-3.75711300	3.21311200
H	-0.82340400	-2.99329200	3.11186700
C	1.23418100	-1.09543200	3.17683800
H	0.46656600	-0.98222000	3.94650600
H	2.09928400	-1.58960800	3.63904800
H	1.52439200	-0.10091500	2.83174400

6-H

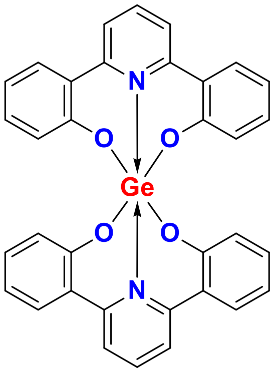

G= - 3794.023 a.e.

Ge	0.11106700	0.02636600	-0.06377700
O	-1.90758400	-2.93475700	0.88408700
O	-0.04139500	1.83636400	0.28648800
O	0.25629400	-0.23625000	1.83538900
O	0.06903500	0.44513400	-1.90450400
N	-2.11408800	0.01203600	0.02869000
N	2.16232900	0.18262900	-0.14054100
C	-2.79596400	-0.87338800	-0.73863800
C	-2.78299600	1.01336500	0.66013700
C	-4.16394000	-0.73980500	-0.96986000
C	-4.16437800	1.18078700	0.45165400
C	-4.85480000	0.31426600	-0.37818000
C	2.87537800	-0.66314300	0.65832200
C	2.77749400	1.05165500	-0.99192800
C	4.27310100	-0.60256100	0.66181300
C	4.18194800	1.08503400	-1.03823300
C	4.92544100	0.27282900	-0.19780800
C	-0.72998300	2.31276500	1.32620400

C	-2.06968000	1.92861400	1.56959900
C	-2.74103500	2.49278900	2.67083200
C	-2.12837000	3.42456000	3.49900900
C	-0.81437900	3.82352900	3.22572000
C	-0.12396000	3.27043300	2.15581300
C	-1.67067900	-3.09454300	-0.45357100
C	-1.03838500	-4.22419900	-0.97576000
C	-2.09472100	-2.06079400	-1.30836300
C	-0.82447600	-4.32903200	-2.35075200
C	-1.88125900	-2.18885400	-2.68233600
C	-1.24805600	-3.31528700	-3.20960700
C	0.69641600	1.52779800	-2.35553300
C	0.07104700	2.29910800	-3.35392700
C	0.66905400	3.44314000	-3.85911800
C	1.91584800	3.85860100	-3.37300400
C	2.55828800	3.09018300	-2.41329200
C	1.98936000	1.90759600	-1.89720600
C	0.93437300	-1.32720100	2.15036500
C	0.39841600	-2.22892100	3.09612500
C	2.17726500	-1.63718700	1.52934300
C	1.06071600	-3.40623200	3.42101700
C	2.81559200	-2.84573700	1.87158900
C	2.27075000	-3.73134500	2.79217200
H	-4.66712800	-1.47104500	-1.59238300
H	-4.67040400	2.01427300	0.92282700
H	-5.91838600	0.44936000	-0.55494700

H	4.83384700	-1.22923700	1.34343900
H	4.67693900	1.72970300	-1.75234800
H	6.01115100	0.31297000	-0.21781600
H	-3.75377300	2.17063500	2.89661400
H	-2.66487800	3.83336400	4.35012300
H	-0.32381400	4.55646900	3.86153000
H	0.89955800	3.55332800	1.93122300
H	-0.71536000	-5.01464000	-0.30235200
H	-0.32655800	-5.20973800	-2.74761400
H	-2.18472800	-1.37686300	-3.33627300
H	-1.07612800	-3.39207400	-4.27890300
H	-0.90604200	1.97122500	-3.69529800
H	0.15717400	4.02848400	-4.61917000
H	2.37611500	4.77197200	-3.73777900
H	3.51389000	3.43200400	-2.02703500
H	-0.54368800	-1.95460000	3.56150000
H	0.63079400	-4.08104900	4.15779800
H	3.74503800	-3.11142300	1.37573700
H	2.78114100	-4.66180200	3.02204700
H	-1.21585100	-3.38333600	1.39955700
H	0.17679600	-1.48279500	-0.35384600

6-Me

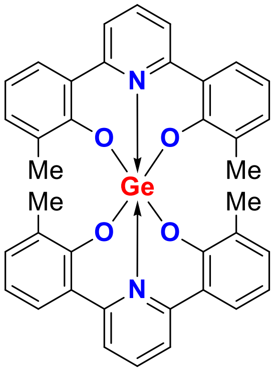

G= - 3951.193 a.e.

Ge	-0.04244300	0.00247000	0.12649500
O	-1.66150400	0.37136300	-0.70930200
O	1.55602700	-0.33073200	1.01248000
O	-0.81640200	-1.45411400	0.98413800
O	0.75210600	1.42016800	-0.77509700
N	-0.58774900	1.25507600	1.66500700
N	0.49777500	-1.24283100	-1.42794300
C	-1.36168600	2.35406100	1.39993400
C	-0.16402400	0.98131500	2.93878800
C	-1.75515400	3.19325900	2.45240300
C	-0.49787200	1.85651700	3.98250100
C	-1.30834000	2.95361200	3.74131300
C	-0.30983300	-2.29914700	-1.75819200
C	1.65220400	-0.99292700	-2.12240800
C	0.06826300	-3.15941300	-2.79992800
C	1.99736000	-1.80931300	-3.20895700
C	1.21283800	-2.90316900	-3.53659300
C	1.53577000	-0.76283300	2.27590500

C	0.66709400	-0.19996200	3.23885100
C	0.68020800	-0.73832800	4.54264700
C	1.54328700	-1.76520600	4.88952100
C	2.42987700	-2.27463800	3.93231400
C	2.43742600	-1.79316000	2.62798100
C	-2.01933600	1.63680400	-0.92906300
C	-2.63609000	1.95526800	-2.15159200
C	-1.82577000	2.65465000	0.03326200
C	-3.00841300	3.25637400	-2.44422500
C	-2.19202700	3.97330900	-0.31273900
C	-2.76955500	4.30200500	-1.53356200
C	2.01862200	1.31870100	-1.17907700
C	2.86217800	2.43355900	-1.03195500
C	4.19153200	2.37629200	-1.41529100
C	4.74315200	1.19789400	-1.95099300
C	3.89408300	0.11105000	-2.12582000
C	2.52736600	0.14137600	-1.77401800
C	-1.71013400	-2.20624600	0.34186300
C	-2.83367900	-2.66256300	1.05319200
C	-1.55135900	-2.57883300	-1.01327100
C	-3.80710500	-3.42756000	0.43351100
C	-2.57941700	-3.33454100	-1.61765600
C	-3.71023500	-3.75944500	-0.93040400
H	-2.42293600	4.01952400	2.25052700
H	-0.10438900	1.67411900	4.97321200
H	-1.59044300	3.61814300	4.55339500

H	-0.53359200	-4.03260600	-3.01118300
H	2.87219700	-1.56778600	-3.79723400
H	1.49271500	-3.55316400	-4.36111000
H	-0.02393900	-0.37100500	5.28245400
H	1.52624800	-2.17584900	5.89477600
H	3.11114200	-3.07916600	4.20134300
H	-2.79156500	1.14679600	-2.85904500
H	-3.47078300	3.47655700	-3.40459400
H	-1.98508800	4.77847000	0.38649900
H	2.43551000	3.33002200	-0.59313700
H	4.82535700	3.25020300	-1.27716100
H	4.31628200	-0.80880800	-2.52071000
H	-2.91287900	-2.37896300	2.09795400
H	-4.67290100	-3.75613200	1.00532000
H	-2.50883300	-3.56899500	-2.67596300
C	3.33948400	-2.35620100	1.56100500
H	2.74898300	-2.73687500	0.71955900
H	4.00223800	-1.58642300	1.14840500
H	3.95467400	-3.17144700	1.95473200
C	-4.80457600	-4.54267100	-1.61785300
H	-4.99426600	-5.49904600	-1.11399200
H	-5.75376500	-3.99117700	-1.62457900
H	-4.54495100	-4.76270400	-2.65865200
C	-3.12488600	5.72934900	-1.88017200
H	-2.56586000	6.08444900	-2.75558100
H	-4.19129200	5.83397300	-2.11795500

H	-2.90298100	6.40876400	-1.05057300
C	6.20797700	1.12217400	-2.31464100
H	6.48369800	1.89481200	-3.04375700
H	6.85078800	1.26733600	-1.43674700
H	6.46142900	0.15026000	-2.75073900

II

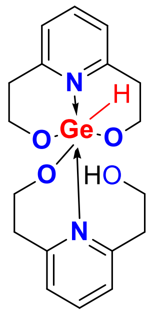

G= - 3184.206 a.e.

Ge	-0.09933100	-0.05675800	-0.10827800
O	-0.48712800	-1.55297100	0.91638500
O	0.91653000	3.70899000	-1.11975700
O	0.23577600	0.92471500	1.48108400
O	0.00205100	-1.07482900	-1.66628300
C	0.52326600	-2.03805900	1.75652400
C	1.75701600	-2.50237100	0.96384000
C	1.91320300	2.93963900	-0.45943700
C	2.43653900	1.79638800	-1.37163400
N	2.16653300	-0.30681100	-0.09784300
N	-2.28774000	0.12091300	-0.14929400
C	3.00699700	0.62307700	-0.61870700
C	2.68219600	-1.40234600	0.50749500
C	4.39248600	0.50134600	-0.48309000
C	4.06190000	-1.55618900	0.67491800
C	4.93065400	-0.58985000	0.18618600
C	-2.89483900	0.84329300	0.82475500
C	-3.02628000	-0.60046300	-1.02674100
C	-4.28283200	0.81449000	0.97441500

C	-4.41780500	-0.65719900	-0.90331600
C	-5.05396400	0.04368900	0.11278100
H	0.12025400	-2.90230800	2.30437600
H	0.83294000	-1.28056700	2.49181100
H	1.39105300	-3.03508300	0.07657300
H	2.34116300	-3.21402700	1.55892600
H	1.53228300	2.52323000	0.47988200
H	2.72783200	3.63219400	-0.22243500
H	3.20298000	2.19001400	-2.04816100
H	1.61383000	1.44381900	-1.99653200
H	5.03082900	1.26581000	-0.91401500
H	4.43688800	-2.44000100	1.18039000
H	6.00541700	-0.69419600	0.30803100
H	-4.74313900	1.39795300	1.76479900
H	-4.98739800	-1.24941100	-1.61183900
H	-6.13442100	0.00366900	0.22155500
C	-1.02545700	-1.99075100	-1.91113800
C	-2.36076200	-1.28444300	-2.19897300
C	-0.83210000	1.05521300	2.37029600
C	-2.06129100	1.72811000	1.71928300
H	-1.15837900	-2.68826000	-1.07269300
H	-0.76313200	-2.57729700	-2.80485100
H	-3.07792900	-1.99393300	-2.62959500
H	-2.16113800	-0.52308700	-2.96683900
H	-0.50920600	1.69324600	3.20669100
H	-1.14169400	0.08473000	2.78983000

H	-1.68289800	2.57350000	1.12826600
H	-2.71769900	2.14088500	2.49394800
H	0.15194100	3.11000900	-1.19478700
H	-0.11471800	1.26796000	-0.94466100

Molecular orbitals of the model compounds

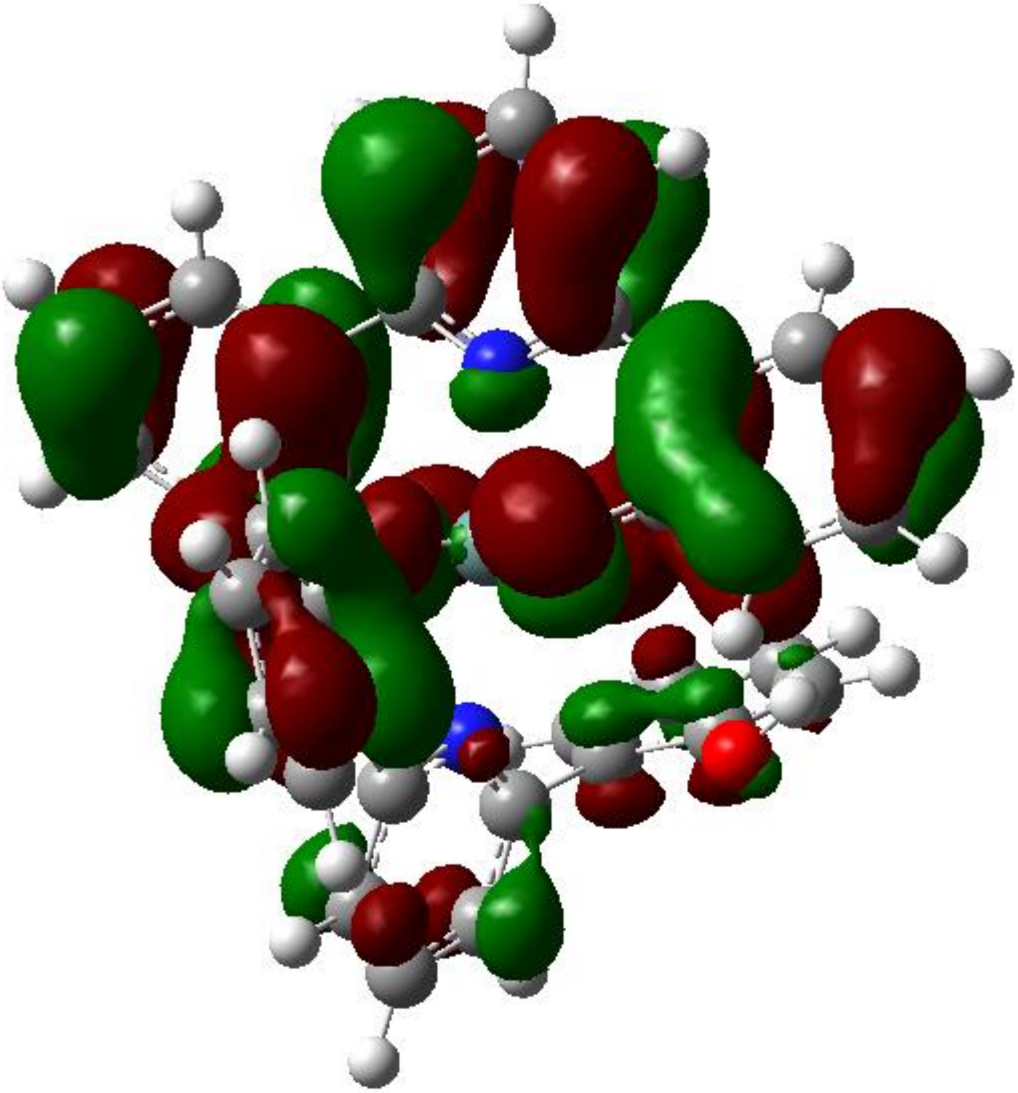

Figure S1. HOMO orbital of compound **6-H**.

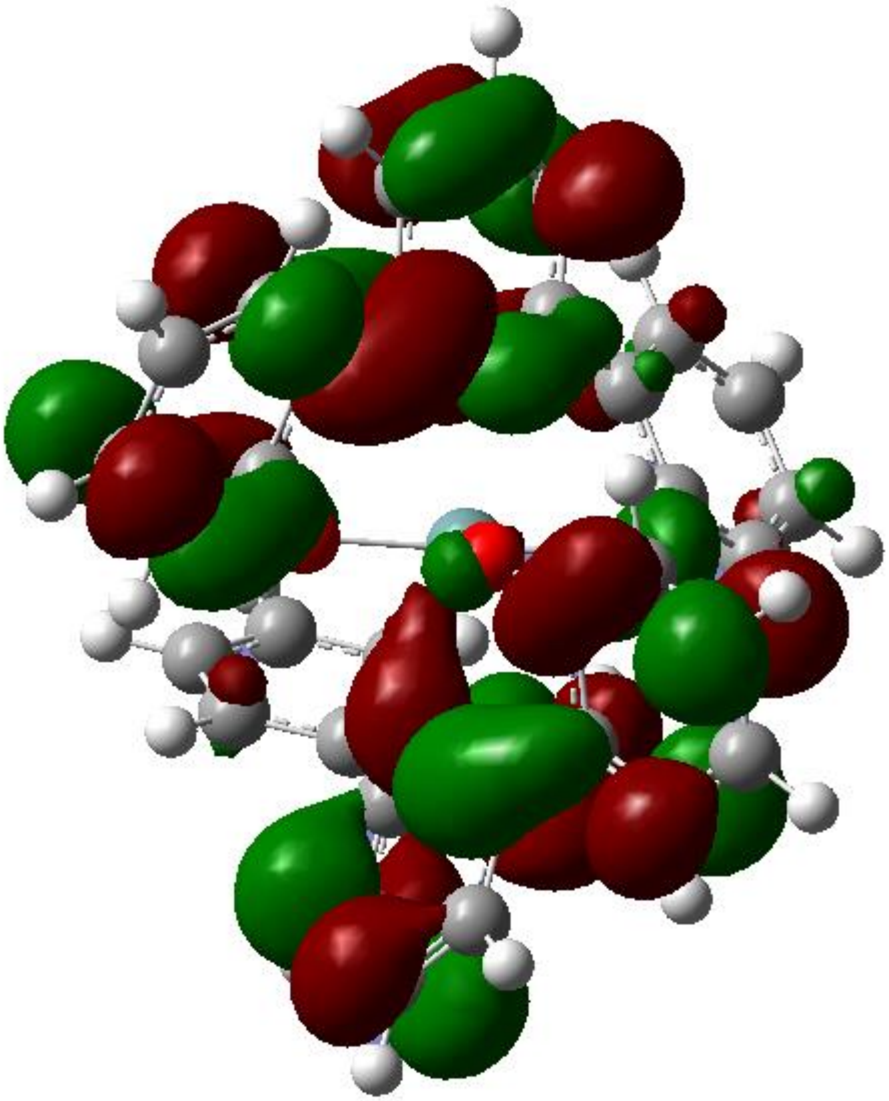

Figure S2. LUMO orbital of compound **6-H**.

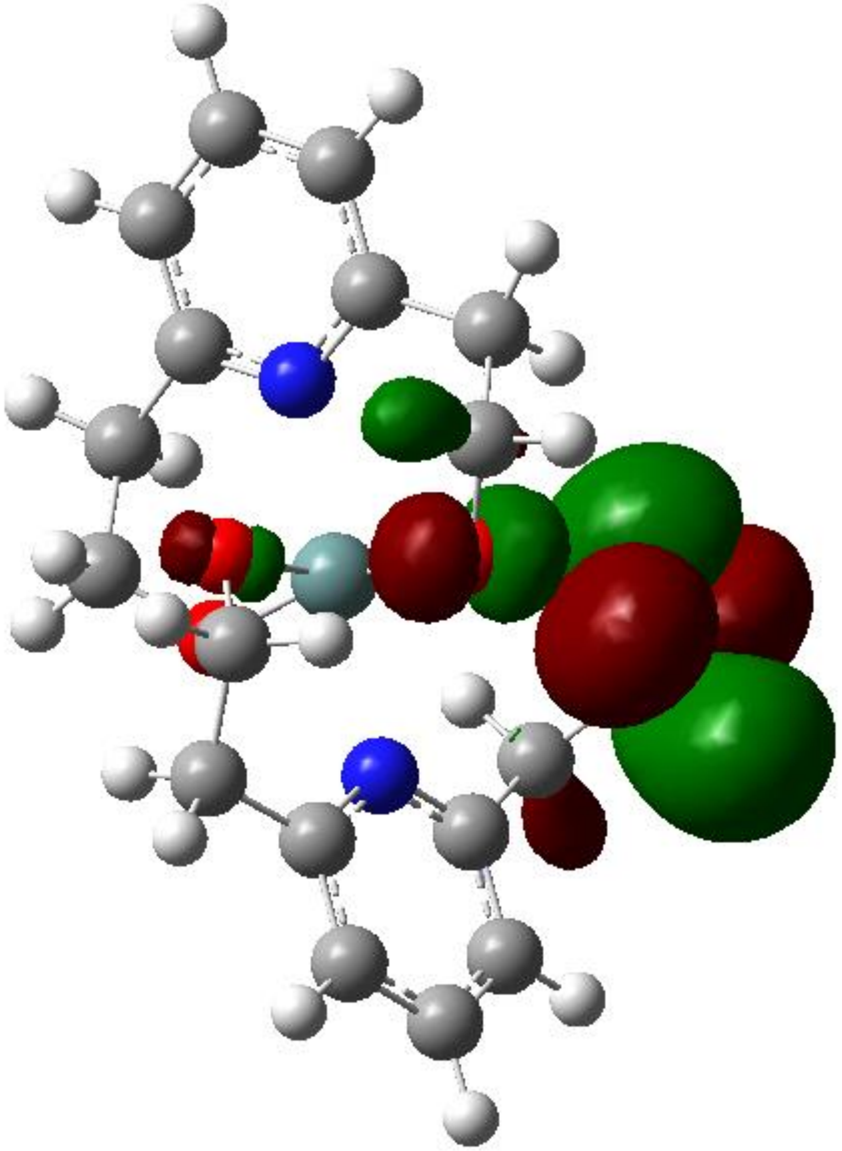

Figure S3. HOMO orbital of compound **II**.

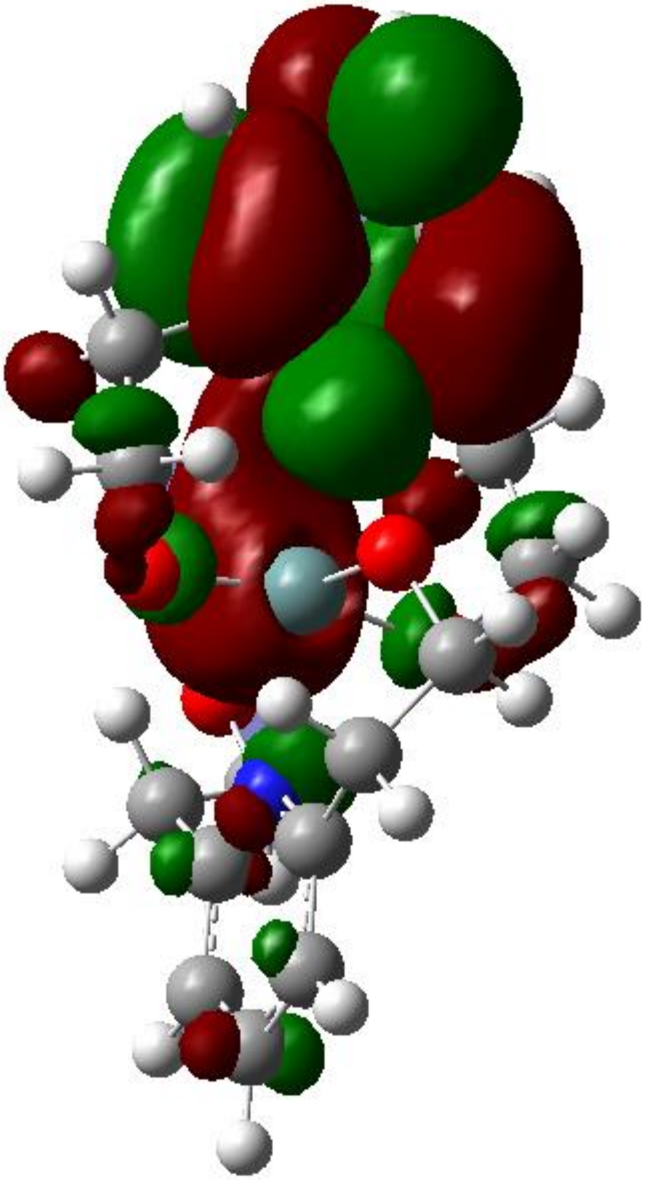

Figure S4. LUMO orbital of compound **II**.
